# Immunohistochemical Localization of Endothelin- 1 and Endothelin A Receptor in Human Primary Tooth Enamel Organ

**DOI:** 10.30476/dentjods.2022.95201.1845

**Published:** 2023-09

**Authors:** Soussan Irani, Shohreh Alimohammadi, Tahmine Najafian

**Affiliations:** 1 Dept. of Oral Pathology, Dental Faculty, Dental Research Centre, Hamadan University of Medical Sciences, Hamadan, Iran. Lecturer at Griffith University, Gold Coast, Australia; 2 Gynecologist and Perinatologist, Hamadan University of Medical Sciences, Hamadan, Iran; 3 Dentist, Private Clinic, Hamadan, Iran

**Keywords:** Embryology, Enamel organ, Endothelin-1, Odontogenesis

## Abstract

**Statement of the Problem:**

Enamel organ (EO) is an ectodermal derived structure, which is involved in the different aspects of tooth development. Tooth development shares the same regulatory molecules and genes expressed in other developing organs. Endothelin- 1 (ET-1) and Endothelin A receptor (ETAR), (ET-1/ETAR) axis, are involved in differentiation of embryonic stem cells and organ development.

**Purpose:**

The present study aimed to investigate the ET-1 and ETAR expression profiles during the development of human primary tooth EO with the relatively large sample size.

**Materials and Method:**

In this experimental study, 33 human fetuses aged from 13 to 23 weeks (3 samples from each fetal age) were collected. The samples were divided into three age groups (<16 weeks, <19 weeks, ≥19 weeks) and cut for hematoxylin and eosin (H&E) and immunohistochemistry (IHC) staining. A two-way ANOVA test was conducted to examine the expression levels of ET-1 and ETAR in different layers of human primary tooth EO. The statistical significance was assumed at p ≤ 0.05.

**Results:**

There were statistically significant differences between the expression levels of ET-1/ETAR axis in the four-layered human primary tooth EO in different fetal ages (13-23 weeks). Besides, there were significant differences between the expression levels of ET-1/ETAR axis in all layers of human primary enamel organ and types of teeth.

**Conclusion:**

Due to the profile of expression of ET-1/ETAR axis, it can be concluded that this axis contributes to the differentiation of all human primary EO layers and secretion of enamel. ET-1/ETAR axis is one of the signaling molecules, which may have crucial roles in tooth development.

## Introduction

The pharyngeal arches are precursors for structures in vertebrate embryos [ 1 ]. A variety of genes, signaling pathways, and growth factors regulate the craniofacial development. In addition, environmental factors and teratogenic agents are critical during morphogenesis and histo-differentiation stages within embryo [ 1 ]. 

Tooth development starts with the formation of dental placodes. From this point, tooth development continues through three stages including bud stage, cap stage, and bell stage [ 2 ]. Enamel organ (EO) is an ectodermal derived structure involved in the different aspects of tooth development [ 3 ]. EO is reported to be a stem cell reservoir [ 4 ]. The coronal part of EO differentiates into four layers including inner enamel epithelium (IEE), stratum intermedium (SI), stellate reticulum (SR), and outer enamel epithelium (OEE) [ 5 ]. In a developing tooth, the shape and pattern depend on epithelial-mesenchymal interactions, which are modulated by several signaling pathways [ 6 ]. Tooth development also shares the same regulatory molecules and genes expressed in other developing organs [ 7 ]. Any malfunction and mutation result in congenital defects as well as development of cysts and tumors, which appear at any time during life in head and neck area [ 6 - 8 ]. 

Endothelins (ETs) are composed of three structurally similar 21-amino acid peptides, including endothelin-1 (ET-1), endothelin-2 (ET-2), and endothelin-3 (ET-3) [ 9 ]. ET-1 is the most common circulating form of ETs. Different cell types such as endothelial cells and vascular smooth muscle cells produce ET-1 [ 10 ]. ET-1 mediates a number of functions (vasoconstriction, pain, and inflammation) by binding to specific cell surface receptors, namely endothelin A receptor (ETAR) and endothelin B receptor (ETBR) [ 9 - 10 ]. ETBR is expressed on vascular smooth muscle cells, endothelial cells, macrophages, and platelets while ETAR is found on vascular smooth muscle cells [ 9 - 10 ]. ET-1 is involved in embryonic stem cell differentiation and organ development [ 11 - 12 ]. In addition, ET-1 has mitogenic properties [ 13 ]. Interestingly, ET-1 is expressed in the pharyngeal arch epithelium while ETAR is expressed on the pharyngeal arch ectomesenchymal cells. These findings may reflect the influence of ET-1/ETAR axis in development of pharyngeal arches [ 14 ]. There are only few animal studies regarding the role of ET-1 and its receptors in odontogenesis [ 15 - 16 ]. Therefore, the role of ET-1/ETAR axis is not yet clear in the development of human teeth. The present cross sectional study aimed to investigate the ET-1 and ETAR expression profiles during the development of human primary tooth EO with the relatively large sample size. 

## Materials and Method

### Sample collection and staining method

The Ethics Committee approved this study (approval No.IR.UMSHA.REC.1398.043). Dilation and curettage was done for pregnancies under 13 weeks’ gestation, and the fetuses over 23 weeks were buried. Hence, only human fetuses aged between 13 weeks to 23 weeks (3 samples for each fetal age) were collected. After obtaining parents’ consent, the head of each sample was cut and placed in a fixative for 2 days. After decalcification with dilute nitric acid (5%), the samples were embedded in paraffin. The blocks were cut for hematoxylin and eosin (H&E) staining. Then, the sections of 4 μm were cut and processed for immunohistochemistry (IHC) according to the previous studies [ 17 ]. Antibodies used in the IHC assay were anti–ET-1 mouse monoclonal antibody (1:170; ab2786) and anti– ETAR rabbit polyclonal antibody 1:170; ab76259). Placenta tissue was used as a positive control, which is known to express ET-1 and ETAR [ 9 ]. The omission of primary antibodies served as the negative control. According to time line of human tooth development, central incisor tooth germ is in the bell stage of development during the 14^th^ and 15^th^ gestational weeks [ 1 , 15 ]. Besides, ameloblasts appear to function in central incisor tooth germ around the 18th week of gestation [ 16 ]. Therefore, the samples were divided into three age groups including <16 weeks, <19 weeks and ≥19 weeks. The cytoplasmic (ET-1) and cell membrane (ETAR) immunostaining were analyzed. The positive stained cells were counted and the percentage of positive cells was recorded for each EO layer of first primary central incisor and first molar of both jaws in all samples [ 17 ]. 

Statistical analysis was carried out using SPSS (version 20.0; SPSS Inc. Chicago, IL). A two-way ANOVA analysis was conducted to examine the effect of fetal age and types of teeth on the expression levels of ET-1 and ETAR in the different layers of EO. The statistical significance was assumed at *p* ≤ 0.05.

## Results

### Histologic analysis for the expression level of ET-1 in all samples

In this study, 25 samples were males and 8 samples were females. The analyses of ET-1 expression level in the four-layered EO are shown in Table 1.

**Table 1:** Analysis of ET-1 expression in the four layered Enamel Organ

Source of Variation	*df*	Mean square	F	*P* Value
Fetal age (IEE)	2	813.995	423.109	0.000
Types of teeth (IEE)	3	125.908	65.446	0.000
Interaction (IEE)	6	5.151	2.677	0.018
Fetal age(SI)	2	795.041	484.220	0.000
Types of teeth (SI)	3	124.119	75.595	0.000
Interaction(SI)	6	5.148	3.136	0.007
Fetal age (SR)	2	424.899	427.373	0.000
Types of teeth (SR)	3	85.451	85.948	0.000
Interaction (SR)	6	12.392	12.464	0.000
Fetal age (OEE)	2	785.051	552.979	0.000
Types of teeth (OEE)	3	136.160	95.909	0.000
Interaction (OEE)	6	7.650	5.389	0.000

The post-hoc analysis indicated a significant difference between the ET-1 expression level in the IEE layer of mandibular central incisor and mandibular first molar EOs (p<0.001), and between the ET-1 expression level in the IEE layer of mandibular central incisor and maxillary central incisor EOs (*p*<0.001). Moreover, this difference was also significant between the ET-1 expression level in the IEE layer of mandibular central incisor and maxillary first molar EOs (*p*< 0.001). 

Additionally, the post-hoc analysis showed a significant difference between the ET-1 expression level in the SI layer of mandibular central incisor and mandibular first molar EOs (p <0.001), and between the ET-1 expression level in the SI layer of mandibular central incisor and maxillary central incisor EOs (*p*< 0.001). This difference was also significant between the ET-1 expression level in the SI layer of mandibular central incisor and maxillary first molar EOs (*p*< 0.001). 

Furthermore, the post-hoc analysis identified a significant difference between the ET-1 expression level in the SR layer of mandibular central incisor and mandibular first molar EOs (*p*< 0.001), and between the ET-1 expression level in the SR layer of mandibular central incisor and maxillary central incisor EOs (*p*< 0.001). This difference was also significant between the ET-1 expression level in the SR layer of mandibular central incisor and maxillary first molar EOs (p<0.001). Moreover, the post-hoc analysis determined a significant difference between the ET-1 expression level in the OEE layer of mandibular central incisor and mandibular first molar EOs (*p*< 0.001), and between the ET-1 expression level in the OEE layer of mandibular central incisor and maxillary central incisor EOs (*p*< 0.001). This difference was also significant between the ET-1 expression level in the OEE layer of mandibular central incisor and maxillary first molar EOs (*p*< 0.001).

### Histologic analysis for the expression level of ETAR in all samples

Table 2 details the expression level of ETAR in all layers of EO.

**Table 2:** Analysis of Endothelin A receptor (ETAR) expression in the four layered enamel organ

Source of Variation	*df*	Mean square	F	*P* Value
Fetal age (IEE)	2	876.935	460.703	0.000
Types of teeth (IEE)	3	147.967	77.735	0.000
Interaction (IEE)	6	4.205	2.209	0.047
Fetal age(SI)	2	791.450	468.044	0.000
Types of teeth (SI)	3	120.451	71.232	0.000
Interaction(SI)	6	5.536	2.682	0.018
Fetal age (SR)	2	393.568	347.266	0.000
Types of teeth (SR)	3	68.561	60.495	0.000
Interaction (SR)	6	21.255	18.754	0.000
Fetal age (OEE)	2	760.552	673.828	0.000
Types of teeth (OEE)	3	136.790	121.192	0.000
Interaction (OEE)	6	8.828	7.821	0.000

The post-hoc analysis distinguished a significant difference between the ETAR expression level in the IEE layer of mandibular central incisor and mandibular first molar EOs (*p*< 0.001), and between the ETAR expression level in the IEE layer of mandibular central incisor and maxillary central incisor EOs (*p*< 0.001). Moreover, this difference was also significant between the ETAR expression level in the IEE layer of mandibular central incisor and maxillary first molar EOs (*p*< 0.001).

 Furthermore, the post-hoc analysis indicated a significant difference between the ETAR expression level in the SI layer of mandibular central incisor and mandibular first molar EOs (*p*< 0.001), and between the ETAR expression level in the SI layer of mandibular central incisor and maxillary central incisor EOs (*p*< 0.001). This difference was also significant between the ETAR expression level in the SI layer of mandibular central incisor and maxillary first molar EOs (*p*< 0.001).

**Figure 1:**
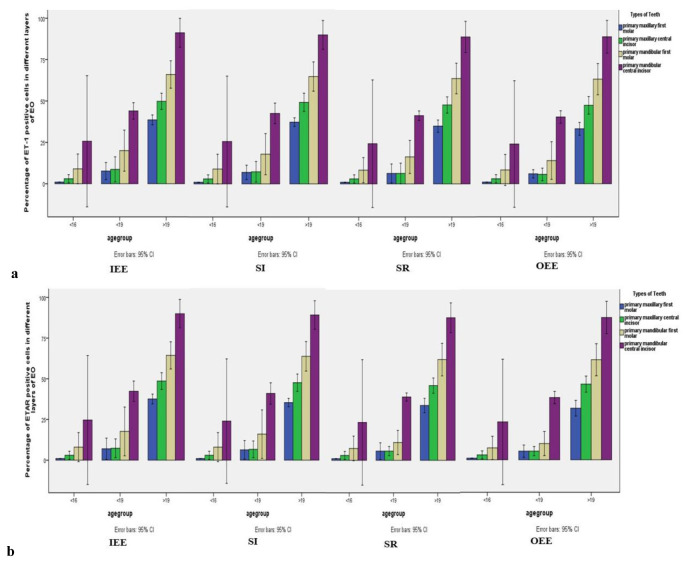
Histograms depicting the percentage of Endothelin- 1 (ET-1), **a:** Endothelin A receptor (ETAR), **b:** Expressions in the different layers of enamel organ (EO) in all fetal ages

 In addition, the post-hoc analysis revealed a significant difference between the ETAR expression level in the SR layer of mandibular central incisor and mandibular first molar EOs (*p*< 0.001), and between the ETAR expression level in the SR layer of mandibular central incisor and maxillary central incisor EOs (*p*< 0.001). This difference was also significant between the ETAR expression level in the SR layer of mandibular central incisor and maxillary first molar EOs (*p*< 0.001). Moreover, the post-hoc analysis found a significant difference between the ETAR expression level in the OEE layer of mandibular central incisor and mandibular first molar EOs (*p*< 0.001), and between the ETAR expression level in the OEE layer of mandibular central incisor and maxillary central incisor EOs (*p*< 0.001). Likewise, this difference was significant between the ETAR expression level in the OEE layer of mandibular central incisor and maxillary first molar EOs (*p*< 0.001) (Figure 1a-b). 

## Discussion

**Figure 2:**
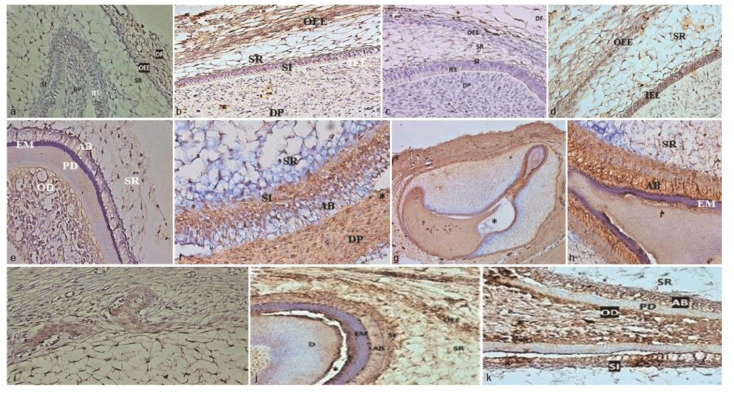
Expression patterns of Endothelin- 1 (ET-1) in the human primary enamel organ (EO) and nearby structures in different fetal ages (A-K) (100 × or 400 ×), **a:** 13-week fetus, **b:** 14-week fetus, **c:** 5-week fetus, **d:** 17-week fetus, **e:** 18-week fetus, **f:** 19-week fetus, **g:** 20-week fetus, **h:** 21-week fetus, **i:** 22-week fetus, **j:** 23-week fetus (mandibular central incisor), **k:** 23-week fetus (maxillary central incisor)

**Figure 3:**
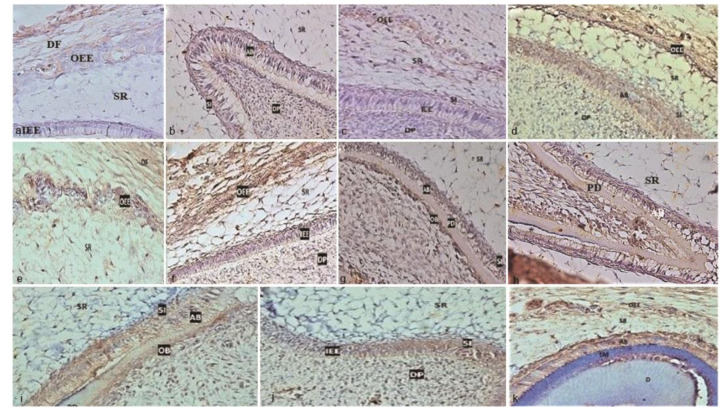
Expression patterns of Endothelin A receptor (ETAR) in human primary enamel organ (EO) and nearby structures in different fetal ages (A-K) (400×), **a:** 14-week fetus, **b:** 15-week fetus, **c:** 16-week fetus, **d:** 17-week fetus., **e:** 18-week fetus, **f:** 19-week fetus, **g:** 20-week fetus (mandibular first molar), **h:** In a 20-week fetus (mandibular central incisor), **i:** In a 21-week fetus, **j:** In a 22-week fetus, **k:** In a 23-week fetus (AB: Ameloblast, D: Dentin, DF: Dental follicle, DP: Dental papilla, EM: Enamel matrix, OD: Odontoblast; PD, predentin

The ET-1/ETAR axis is critical for development and growth in a variety of organs [ 8 , 18 ]. We herein report the expression level of ET-1/ETAR axis in the different layers of human primary EO. Consistent with well-established functions of ET-1/ETAR axis, the cell proliferation of EO is tightly linked to ET-1/ETAR axis [ 14 ]. In this aspect, our findings have demonstrated the immunohistochemical localization of the ET-1/ETAR axis in all layers of human primary EO (Figure 2-3).

In addition, the present study has indicated the elevated expression level of ET-1/ETAR from the bud stage onward in all four layers of human primary EO. Additionally, data analysis in this study, showed a striking increase in the number of positive cells in the human primary EO layers with increase of fetal age. These findings may indicate the role of ET-1/ETAR axis in cell proliferation and differentiation of the different layers of human primary EO. A study detailing the investigation on developing rat tooth has found high densities of ET-1 and ETAR immune-reactivity in ameloblasts [ 12 ]. 

A research, using p63, has indicated stem cells in the SI and SR layers and ameloblasts [ 19 ]. Another study has shown the sonic hedgehog expression in the SI cell layer which confirms the role of the SI cells as the primary early stem cells of the EO [ 20 ]. In addition, it has been shown that the SI cells interact with the IEE cells and ameloblasts. The SI cells are required for the differentiation of ameloblasts [ 3 ]. The highest expression levels of Notch1, 2 and 3 in the SI cells can also prove the presence of stem cells in the SI layer [ 21 ]. It is suggested that the SI stem cells may originate from the SR or ameloblast lineages [ 19 , 22 ]. It has been established that ET-1/ETAR axis plays a crucial role in the differentiation of stem cells [ 23 - 24 ]. Besides, ET-1/ETAR axis has been proven to activate mitogenic pathways in different cell types such as osteoblasts and endothelial cells [ 23 ]. Immunostaining of ET-1/ETAR axis in the current study, may confirm the role of ET-1/ETAR axis in the differentiation of stem cells and proliferation of different cell types of EO.

Previous investigations have shown the production of high amounts of alkaline phosphatase by the SI cells [ 25 - 26 ]. Both the SI and IEE cells function synergistically and can be considered as a single functional unit responsible for enamel formation [ 27 ]. 

These findings may signify the role of SI cells in the differentiation of IEE layer (ameloblasts) and enamel formation [ 20 , 22 , 28 ]. On the other hand, the ET-1/ETAR axis involves in Ca^2+^ transportation and induces an increase in the intracellular Ca^2+^ concentration [ 24 , 29 ]. In our study, there was a gradual increase in ET-1 and ETAR expression level with increase of fetal age in the SI and IEE cell layers (ameloblasts). Therefore, it can be assumed that the ET-1/ETAR axis contributes to enamel formation.

A previous investigation has speculated that ET-1, secreted by endothelial cells, may promote the reciprocal functions of endothelial cells and dental pulp stem c-ells [ 24 ]. Additionally, Aida *et al*. [ 30 ] have revealed the increased expression level of VEGF in the IEE cells and ameloblasts. They concluded that VEGF expression is associated with the differentiation of IEE cells to ameloblasts via activation of map kinase leading to cell proliferation [ 30 ]. Besides, the expression of VEGF in the IEE, SR and OEE layers of EO has been reported [ 31 - 32 ]. On the other hand, ET-1 enhances VEGF expression and angiogenesis via ETAR [ 18 ]. These reports may indicate that the ET-1/ETAR axis controls the expression of VEGF and angiogenesis in the primary tooth EO. 

Earlier investigations have revealed that the blood capillaries are initially juxtaposed to the OEE layer [ 33 ]. Besides, the OEE layer is not a continuous layer; therefore, the capillaries penetrate the EO through these gaps. These findings suggest that when the tooth germ is fully developed, the process of vascularization of EO occurs [ 33 ]. Our study confirms the previous results, demonstrating that the capillaries are found next to the OEE layer and penetrate to this layer. 

However, results from this study suggest further study with larger sample size for better understanding the role of ET-1/ETAR axis in tooth development.

## Conclusion

This study sheds a new light on the expression level of ET-1/ETAR axis in the human primary tooth EO as a four-layered tissue complex and a stem cell niche. Due to the profile of expression of ET-1/ETAR axis, it can be concluded that aforementioned axis contributes to proliferation and differentiation of the four-layered primary enamel organ via different mechanisms such as angiogenesis. Therefore, ET-1/ETAR axis involves in the secretion of tooth enamel. ET-1/ETAR axis is one of the signaling molecules that EO might use to exert roles in tooth development. Besides, our study may prove that the capillaries close to the OEE layer gradually penetrate to the primary tooth EO. Further studies with larger sample size are required for better understanding of the role of ET-1/ETAR axis in tooth development.

## Acknowledgement

The authors would like to thank Hamadan University of Medical Sciences for the financial support of the project.

## Conflict of Interest

The authors declare that they have no conflicts of interests.
